# Intestinal PPARδ protects against diet-induced obesity, insulin resistance and dyslipidemia

**DOI:** 10.1038/s41598-017-00889-z

**Published:** 2017-04-12

**Authors:** Marcela Doktorova, Irene Zwarts, Tim van Zutphen, Theo H. van Dijk, Vincent W. Bloks, Liesbeth Harkema, Alain de Bruin, Michael Downes, Ronald M. Evans, Henkjan J. Verkade, Johan W. Jonker

**Affiliations:** 1 Section of Molecular Metabolism and Nutrition, Department of Pediatrics, University of Groningen, University Medical Center Groningen, Hanzeplein 1, 9713 GZ Groningen, The Netherlands; 2 Department of Laboratory Medicine, University of Groningen, University Medical Center Groningen, Hanzeplein 1, 9713 GZ Groningen, The Netherlands; 3 Dutch Molecular Pathology Center, Faculty of Veterinary Medicine, Utrecht University, Yalelaan 1, 3584 CL Utrecht, The Netherlands; 4 Howard Hughes Medical Institute and Gene Expression Laboratory, Salk Institute for Biological Studies, 10010 North Torrey Pines Road, La Jolla, California 92037 USA

## Abstract

Peroxisome proliferator-activated receptor δ (PPARδ) is a ligand-activated transcription factor that has an important role in lipid metabolism. Activation of PPARδ stimulates fatty acid oxidation in adipose tissue and skeletal muscle and improves dyslipidemia in mice and humans. PPARδ is highly expressed in the intestinal tract but its physiological function in this organ is not known. Using mice with an intestinal epithelial cell-specific deletion of PPARδ, we show that intestinal PPARδ protects against diet-induced obesity, insulin resistance and dyslipidemia. Furthermore, absence of intestinal PPARδ abolished the ability of PPARδ agonist GW501516 to increase plasma levels of HDL-cholesterol. Together, our findings show that intestinal PPARδ is important in maintaining metabolic homeostasis and suggest that intestinal-specific activation of PPARδ could be a therapeutic approach for treatment of the metabolic syndrome and dyslipidemia, while avoiding systemic toxicity.

## Introduction

The prevalence of obesity and related chronic metabolic diseases such as type 2 diabetes, cardiovascular disease and certain types of cancer is increasing worldwide at an alarming rate. Peroxisome proliferator-activated receptors (PPARs) have emerged as key targets for the treatment of these disorders. PPARs constitute a subfamily of the nuclear receptor family of ligand-activated transcription factors. This subfamily consists of three members, PPARα, -β/δ and –γ (NR1C1–3), which are activated by (dietary) lipids, specifically polyunsaturated fatty acids, and have critical functions in lipid metabolism^1^. PPARs are also potent regulators of the inflammatory and immune response by antagonizing the activities of other transcription factors such as members of the nuclear factor-κB (NF-κB) and activator protein-1 (AP-1) families, a process which is named *trans*-repression^2^. PPARα is the molecular target of the fibrate class of lipid-lowering drugs and is primarily expressed in tissues with a high level of fatty acid catabolism such as liver, brown fat, kidney, heart and skeletal muscle where it regulates fatty acid oxidation and apolipoprotein synthesis^3–5^. PPARγ is the molecular target of the thiazolidinedione (TZD) class of insulin-sensitizing drugs and is essential for adipocyte differentiation and fat storage^6^.

PPARδ (also known as PPARβ) is ubiquitously expressed and when activated it promotes fatty acid oxidation, thermogenesis, insulin sensitivity, high density lipoprotein cholesterol (HDLc) levels in plasma and overall energy expenditure^7^. PPARδ deficient mice are prone to obesity and insulin resistance when challenged with a high-fat diet (HFD). Conversely, transgenic expression of a constitutively active form of PPARδ in adipose tissue or skeletal muscle protects mice from diet-induced obesity and regulates muscle fiber type switching, respectively^8, 9^.

Treatment of mice with the high-affinity PPARδ agonist GW501516 increases plasma levels of HDLc and reduces lesion progression in mouse models of atherosclerosis^10, 11^. In obese rhesus monkeys and healthy humans PPARδ agonist administration also increases plasma levels of HDLc and decreases low density lipoprotein cholesterol (LDLc) and triglycerides (TGs)^12, 13^. In addition, PPARδ agonists act as exercise mimetics by transcriptional remodeling of skeletal muscle resulting in oxidative fiber type switch and improved running endurance^14^. Based on these findings a number of small molecule PPARδ agonists, including MBX-8025 (Metabolex) and KD3010 (Kalypsys) are currently under evaluation in clinical trials for dyslipidemia and other aspects of the metabolic syndrome^7, 15, 16^. However, adverse effects, such as the potency of GW501516 to induce cancer in rodent models, and widespread abuse by athletes have complicated their progression into the clinic^17^.

Although PPARδ is abundantly expressed along the entire intestinal tract, its potential role in energy homeostasis in this organ has not been well explored^18^. Daoudi *et al*. showed a role of intestinal PPARδ in the stimulation of post-prandial glucagon-like protein-1 (GLP1) production in enteroendocrine L-cells, resulting in preservation of β-cell morphology and function and, thereby, increased systemic insulin sensitivity^19^. In the present study we evaluated a possible role of PPARδ in the intestine in energy metabolism and the development of metabolic syndrome using mice with an intestinal epithelial specific deletion of the *PPAR*δ gene. Here we show that intestinal PPARδ contributes to the protection against diet-induced obesity and that intestinal PPARδ is required for mediating the increase in plasma levels of HDLc by PPARδ activation. Together, these results suggest targeting of intestinal PPARδ as a potential approach for the therapeutic treatment of dyslipidemia, obesity and insulin resistance, with limited systemic toxicity.

## Results

### Characterization of intestinal epithelial cell (IEC) specific PPARδ knockout (*PPARδ*^*IEC-KO*^) mice

**Figure 1:**
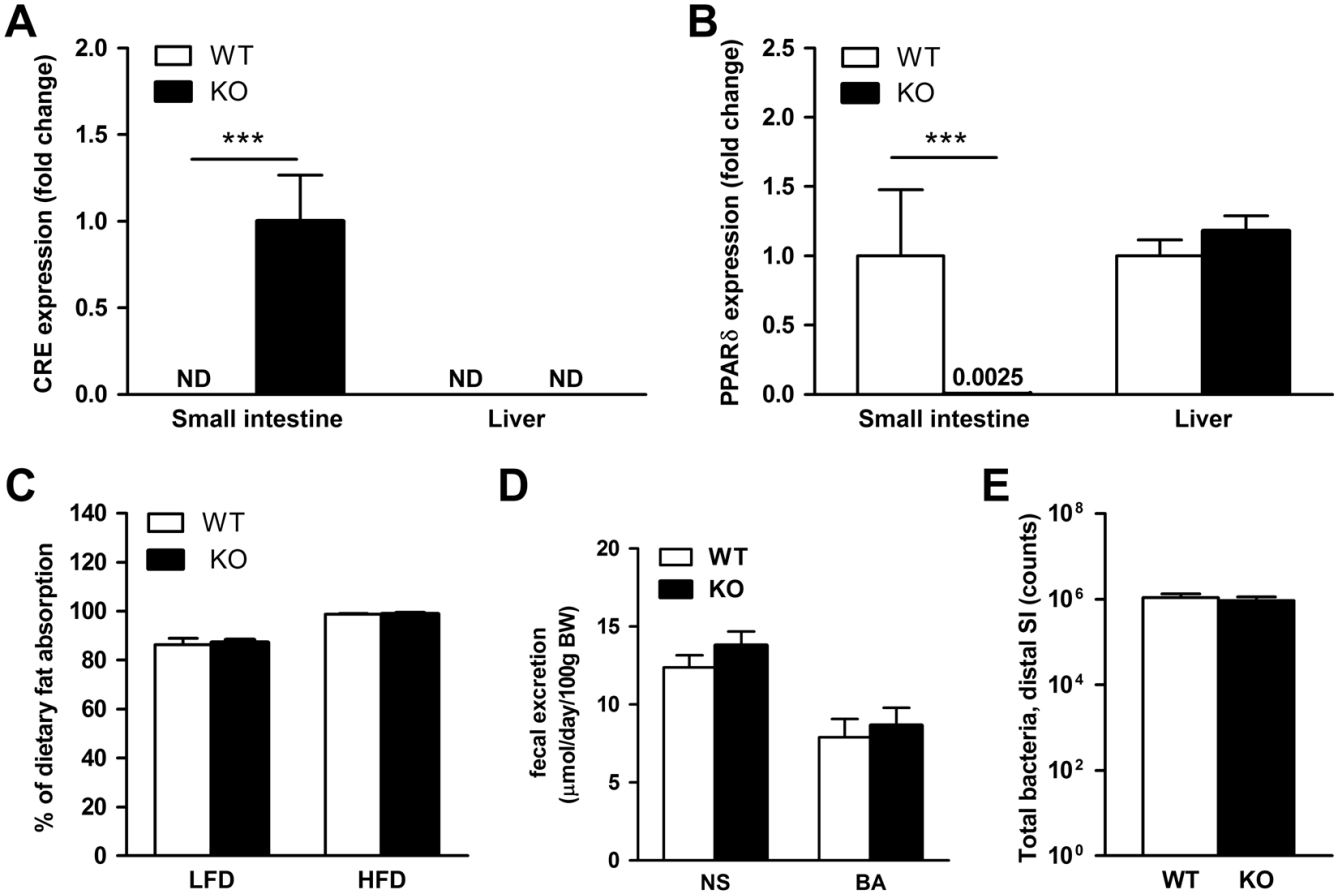
Characterization of mice with an intestinal epithelial specific deletion of the PPARδ gene (*PPAR*δ^*IEC-KO*^). mRNA levels of (**A**) Cre recombinase and (**B**) PPARδ in mucosal scrapings from the small intestine (normalized to 36b4); ND = not detectable; (**C**) Fat balance (% of dietary fat absorption) in mice on a LFD and HFD (45% energy content in fat); (**D**) Fecal excretion of neutral sterols (NS) and bile acids (BA); (**E**) Total bacterial counts in the distal part of the intestine (in intestinal content and mucosa combined) in wild-type and *PPAR*δ^*IEC-KO*^ mice (n = 7–9).

Mice with an intestinal epithelial cell (IEC) specific deletion of PPARδ were generated by cross-breeding mice carrying loxP sites on either side of exon 4 of the *PPAR*δ gene (*PPAR*δ^*lox/lox*^) with transgenic mice expressing Cre recombinase under the control of the IEC-specific villin promoter. Expression of Cre recombinase mRNA was specifically localized to the intestine and absent in the liver of *PPAR*δ^*IEC-KO*^ mice (Fig. 1A). Absence of *PPAR*δ mRNA in small intestinal mucosa was confirmed by qPCR using primers detecting exon 4 of *PPAR*δ (Fig. 1B). Under standard housing conditions *PPAR*δ^*IEC-KO*^ mice displayed no obvious phenotype. They were born at the expected Mendelian ratio, and there were no differences in food intake, body weight, liver weight and plasma and hepatic lipid composition as compared to their wild-type littermates (Table 1). Also, no differences were observed in dietary fat absorption and fecal excretion of neutral sterols (NS) and bile acids (BA) (Fig. 1C,D).

**Table 1:** Animal characteristics and liver composition of wild-type and *PPAR*δ^*IEC-KO*^ mice with and without GW501516 treatment on low fat diet.

	wild-type	*PPAR*δ^*IEC-KO*^	wild-type GW501516	*PPAR*δ^*IEC-KO*^ GW501516
**Food intake (g/24 h)**	3.6 ± 0.5	3.8 ± 0.6	3.9 ± 1.1	3.7 ± 1.1
**Body weight (g)**	26.5 ± 2.2	25.9 ± 2.6	26.2 ± 1.3	25.0 ± 1.8
**Liver weight (% of body weight)**	3.9 ± 0.3	4.1 ± 0.3	4.9 ± 0.4^***^	4.8 ± 0.3^###^
**Liver cholesterol (µmol/g)**	4.2 ± 0.2	4.0 ± 0.3	4.0 ± 0.3	4.1 ± 0.1
**Liver TG (µmol/g)**	7.9 ± 0.9	10.6 ± 2.0	11.1 ± 4.4	7.3 ± 1.4
**Liver phospholipids (µmol/g)**	17.3 ± 1.1	17.3 ± 1.0	16.9 ± 1.0	17.8 ± 0.5
**Plasma TG (mmol/l)**	1.2 ± 0.3	0.9 ± 0.4	1.5 ± 0.4	0.8 ± 0.3
**Plasma cholesterol (mmol/l)**	3.5 ± 1.0	3.5 ± 0.9	4.7 ± 0.6	4.1 ± 0.4

^***^Significantly different from wild-type (p < 0.001); ^###^Significantly different from *PPAR*δ^*IEC-KO*^ (p < 0.001); Values are presented as means ± SD (n = 6–7).

Histopathologic examination of the small intestine (proximal, middle and distal part) revealed no differences in villus length, crypt depth and inflammation scoring between wild-type and *PPAR*δ^*IEC-KO*^ mice. We found a reduction in the number of Paneth cells, specialized crypt cells involved in immunity and production of antimicrobial compounds, in all three sections of the small intestine of *PPAR*δ^*IEC-KO*^ mice, although this did not reach statistical significance (median 1.5 [0.5–3.0] vs 2.0 [1.0–3.0], *p* = 0.13). This observation is in line with a previous study that reported a role for PPARδ in the regulation of Paneth cell differentiation through hedgehog signaling, using whole body PPARδ knockout mice^20^. The latter study also reported changes in the microbial composition, with a decrease in Lactobacilli and an increase in Bifidobacteria^20^. In the current study, however, we did not find any differences in Lactobacilli, Eubacteria or total number of bacteria in the middle and distal part of the small intestine (Fig. 1E, Supplementary Fig. 1), cecum, colon and feces (data not shown) between wild-type and *PPAR*δ^*IEC-KO*^ mice.

### Intestinal PPARδ protects against diet-induced obesity and insulin resistance

**Figure 2:**
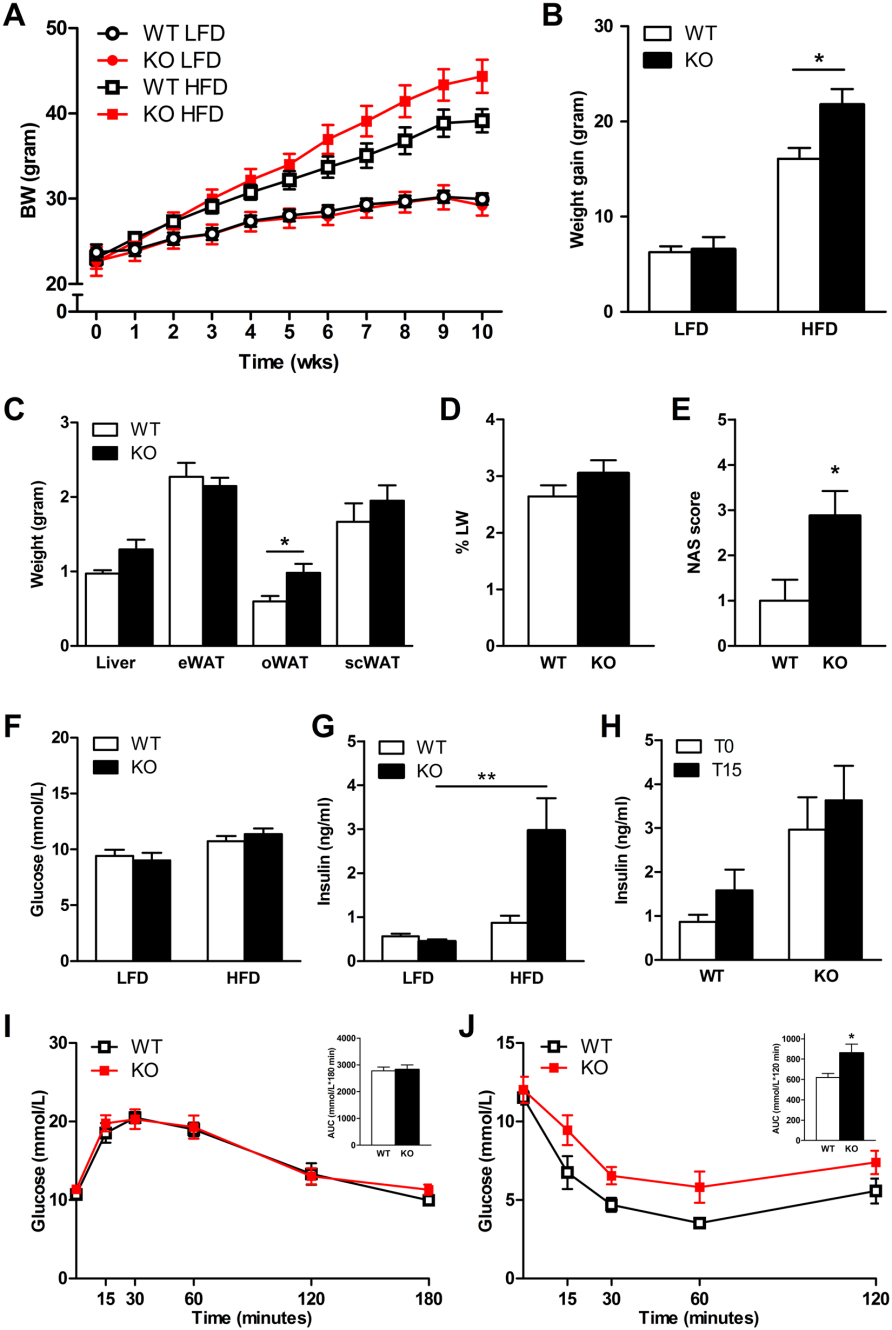
Effect of a HFD on diet-induced obesity, hepatic steatosis and insulin sensitivity in *PPAR*δ^*IEC-KO*^ and wild-type mice. Effect of a HFD challenge on (**A**) Body weight; (**B**) Body weight gain; (**C**) Liver and white adipose tissue (WAT) (epididymal, omental and subcutaneous) weights; (**D**) Liver weight as % of body weight; (**E**) NAFLD activity score (NAS score); (**F**) Fasting blood glucose; (**G**) Fasting blood insulin; (**H**) Glucose stimulated insulin secretion (GSIS, 2 g/kg p.o. glucose); (**I**) Oral glucose tolerance test (OGTT, 2 g/kg p.o. glucose), graph insert showing area under the curve (AUC); (**J**) Insulin tolerance test (ITT, graph insert showing AUC, in *PPAR*δ^*IEC-KO*^ mice and wild-type littermates (n = 7–10).

To determine the role of intestinal PPARδ in the development of metabolic syndrome we challenged *PPAR*δ^*IEC-KO*^ mice and wild-type littermates for 10 wks with a HFD consisting of 60% kcal from fat. Whereas body weight gain during 10 wks on a control low-fat diet (LFD, 10% kcal from fat) was not different between genotypes, *PPAR*δ^*IEC-KO*^ displayed an increased body weight gain in response to HFD as compared to their wild-type littermates (Fig. 2A,B). Further analysis revealed a significant increase was in the amount of omental white adipose tissue (oWAT) in *PPAR*δ^*IEC-KO*^ mice as compared to their wild-type littermates, whereas the weight of epididymal and subcutaneous WAT depots were not different between genotypes. Although liver weight (Fig. 2C) or liver weight as % of body weight (LW%) (Fig.﻿ 2D) and TG content (Table 2) were not different, NAFLD activity score (NAS) based on histological analysis of Hematoxylin & Eosin (H&E) stained liver sections, was significantly increased in *PPAR*δ^*IEC-KO*^ mice from 1.0 ± 1.3 to 2.9 ± 1.6 (*P* < 0.05) as compared to wild-type littermates, respectively (Fig. 2C–E, Supplementary Fig. 2A,B, Supplementary Table 2). *PPAR*δ^*IEC-KO*^ mice on a HFD displayed significantly increased levels of fasting plasma insulin and increased insulin resistance as compared to their wild-type littermates (Fig. 2G,H,J). Fasting plasma glucose levels and oral glucose tolerance on the other hand were not different (Fig. 2F,I). In addition, no differences were observed in food intake, respiratory exchange rate (RER), activity (Table 2, Supplementary Fig. 2C,D) and microbiota in the distal part of the small intestine (data not shown).

**Table 2:** Animal characteristics and liver composition of wild-type and *PPAR*δ^*IEC-KO*^ mice after a LFD (n = 3–8) or HFD (n = 4–10).

	wild-type LFD	*PPAR*δ^*IEC-KO*^ LFD	wild-type HFD	*PPAR*δ^*IEC-KO*^ HFD
**Food intake (g/24 h)**	2.8 ± 0.6	2.3 ± 0.6	2.7 ± 0.5	2.9 ± 0.5
**Body weight (g)**	30.0 ± 2.1	29.2 ± 3.1	39.1 ± 4.3	44.4 ± 6.2
**Liver weight (% of body weight)**	3.6 ± 0.3	2.9 ± 0.5	2.6 ± 0.6^*^	3.1 ± 0.7
**Liver cholesterol (µmol/g)**	6.0 ± 2.3	5.2 ± 0.6	6.4 ± 1.3	5.1 ± 2.0
**Liver TG (µmol/g)**	2.5 ± 2.1	2.6 ± 1.0	6.7 ± 10.1	6.5 ± 10.5
**Liver phospholipids (µmol/g)**	26.6 ± 1.6	25.0 ± 0.6	27.9 ± 2.1	26.7 ± 2.8
**Plasma TG (mmol/l)**	0.7 ± 0.4	0.6 ± 0.2	0.5 ± 0.2	0.5 ± 0.2
**Plasma cholesterol (mmol/l)**	3.3 ± 0.3	2.6 ± 0.9	4.2 ± 0.6	5.0 ± 0.7^###^

^*^Significantly different from wild-type on LFD (p < 0.05); ^###^Significantly different from *PPAR*δ^*IEC-KO*^ on LFD (p < 0.001). Values are presented as means ± SD.

**Figure 3:**
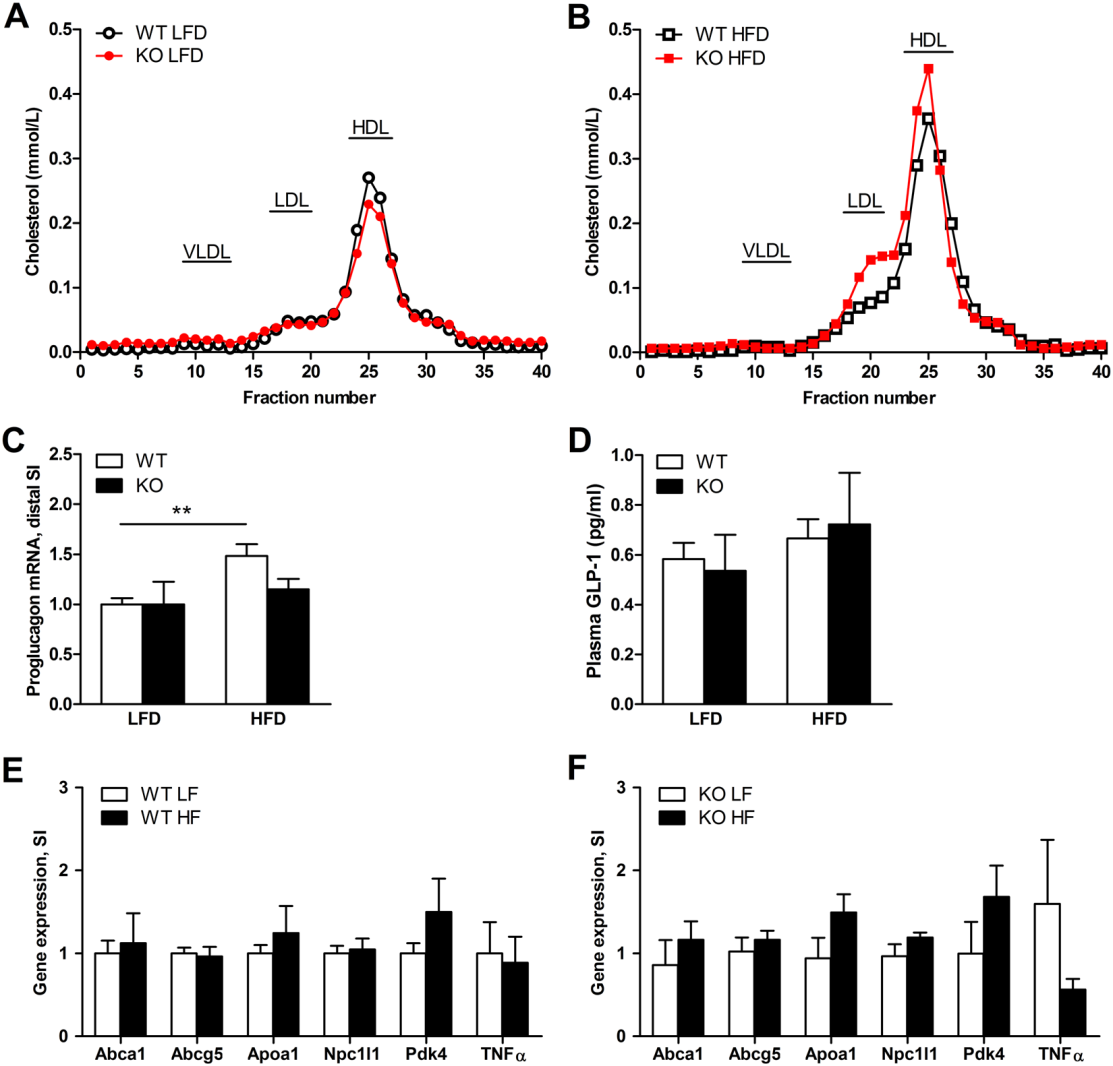
Effect of a HFD on plasma lipoprotein, plasma GLP1 and intestinal gene expression in *PPAR*δ^*IEC-KO*^ and wild-type mice. (**A**,**B**) FPLC Lipoprotein cholesterol profiles of pooled plasma samples from *PPAR*δ^*IEC-KO*^ mice and wild-type littermates fed a (**A**) LFD (n = 4–5) or (**B**) HFD (n = 10); Effect of a HFD challenge on (**C**) mRNA levels of proglucagon in distal small intestine (normalized to 36b4); (**D**) Plasma levels of Active GLP-1 (7–36) amide and GLP-1 (7–37) in wild-type and *PPAR*δ^*IEC-KO*^ mice fed a LFD (n = 4–8) or HFD (n = 10); (**E**,**F**) mRNA levels of genes involved in lipoprotein metabolism and inflammation in the mucosa of the distal small intestine of (**E**) wild-type and (**F**) *PPAR*δ^*IEC-KO*^ mice (normalized to 36b4).

*PPAR*δ^*IEC-KO*^ mice challenged with a HFD displayed increased plasma levels of total cholesterol (Table 2). Further analysis by fast protein liquid chromatography (FPLC) confirmed increased levels of total cholesterol in *PPAR*δ^*IEC-KO*^ mice and showed that this was mainly due to higher levels of LDLc. This difference in lipoprotein profile was not seen on a control LFD (Fig. 3A,B). Levels of proglucagon mRNA in the distal small intestine were significantly increased by a HFD in wild-type mice but not in *PPAR*δ^*IEC-KO*^ mice. However, this difference in mRNA did not result in reduced plasma levels of GLP-1 in *PPAR*δ^*IEC-KO*^ mice at 30 min after a glucose bolus (Fig. 3C,D). Also, no changes in the expression of genes involved in cholesterol transport and the pro-inflammatory cytokine TNFα were observed in the distal small intestine of *PPAR*δ^*IEC-KO*^ mice and wild-type littermates challenged with a HFD (Fig. 3E,F). Together, these results show that intestinal PPARδ protects against HFD induced obesity, insulin resistance and dyslipidemia.

### Role of intestinal PPARδ in the response to treatment with the PPARδ agonist GW501516

To determine the contribution of intestinal PPARδ to the response to treatment with a PPARδ-specific agonist, wild-type and *PPAR*δ^*IEC-KO*^ mice were orally treated for 14 days with GW501516 (3 mg/kg) or vehicle. As previously reported, liver weight was significantly increased by GW501516^21, 22^. However, this increase was also observed in PPARδ^*IEC-KO*^ mice, suggesting that this effect was independent of intestinal specific activation of PPARδ (Table 1). No effect of GW501516 was observed on food intake, body weight, hepatic lipid composition and microbiota in the distal part of the small intestine in *PPAR*δ^*IEC-KO*^ mice as compared to their wild-type littermates (Table 1 and data not shown).

### Intestinal PPARδ is required for the increase in plasma HDLc by GW501516

**Figure 4:**
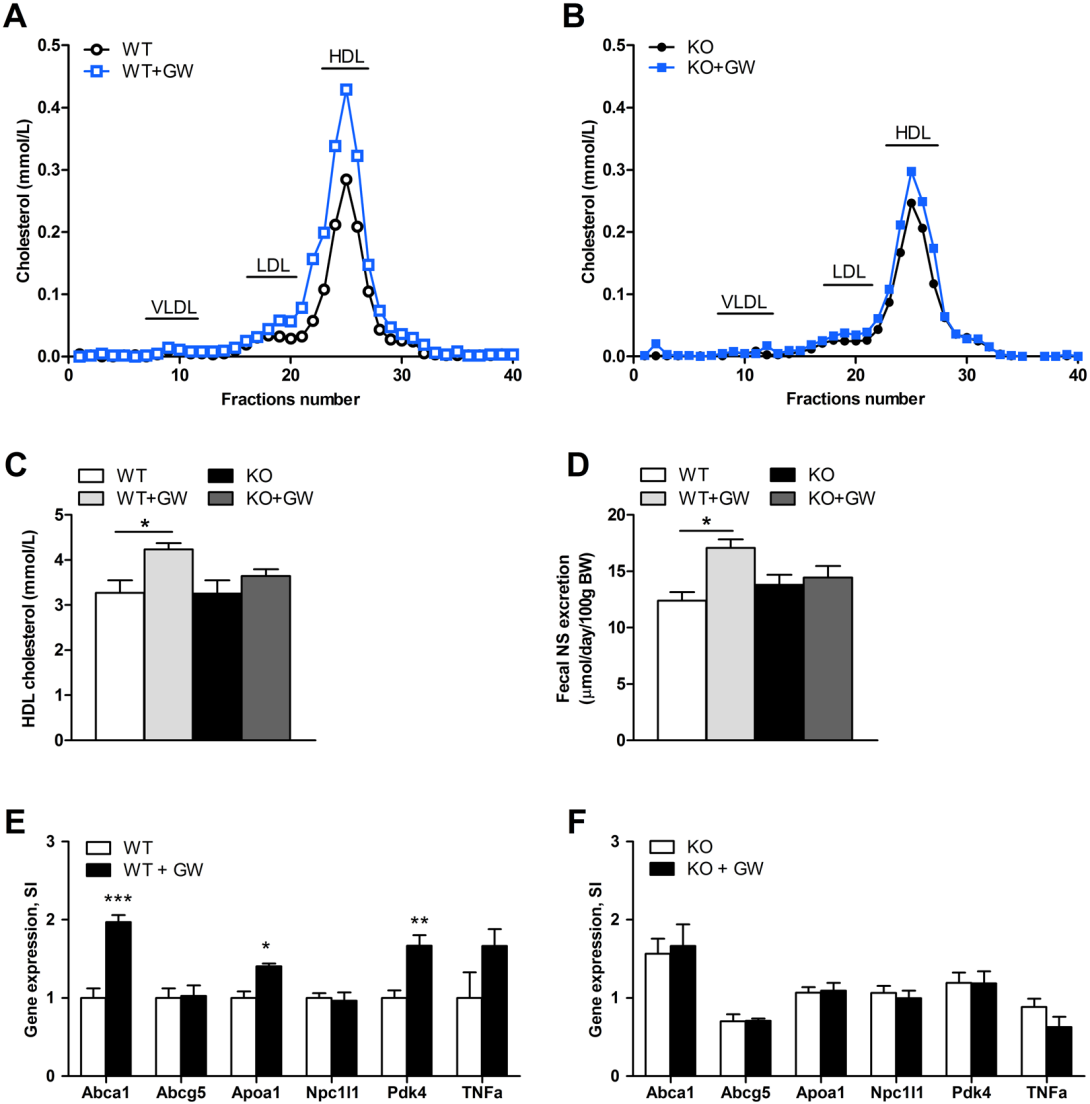
Effect of PPARδ activation on cholesterol metabolism and intestinal gene expression in *PPAR*δ^*IEC-KO*^ and wild-type mice. (**A**,**B**) FPLC Lipoprotein cholesterol profiles of pooled plasma samples from (**A**) wild-type and (**B**) *PPAR*δ^*IEC-KO*^ mice treated with for 14 days with GW501516 or vehicle (n = 7). Plasma levels of (**C**) HDLc; and (**D**) Fecal neutral sterol (NS) excretion in wild-type and *PPAR*δ^*IEC-KO*^ mice (n = 7) treated for 14 days with GW501516 (GW) or vehicle; (**E**,**F**) Levels of mRNA (normalized to 36b4) of genes involved in lipoprotein metabolism and inflammation in the mucosa of the small intestine of (**F**) wild-type and (**G**) *PPAR*δ^*IEC-KO*^ mice treated for 14 days with GW501516 or vehicle (n = 7).

Previously, it has been reported that small molecule agonists of PPARδ can effectively increase plasma levels of HDLc in rodents, primates and humans^12, 23, 24^. It remains unclear, however, to what extent the intestine contributes to this effect. FPLC analysis showed that plasma levels of HDLc were increased by approximately 50% by GW501516 treatment in wild-type mice but not in *PPAR*δ^*IEC-KO*^ mice (Fig. 4A,B). This finding was supported by biochemical analysis of plasma, showing significantly elevated plasma levels of HDLc by GW501516 treatment in wild-type mice but not in *PPAR*δ^*IEC-KO*^ mice (Fig. 4C). In line with earlier findings, the excretion of neutral sterols in the feces was significantly increased by GW501516 in wild-type mice and this effect was not observed in *PPAR*δ^*IEC-KO*^ mice, indicating that activation of intestinal PPARδ is required for the fecal excretion of neutral sterols (Fig. 4D). GW501516 treatment increased the mRNA levels of the known PPARδ targets *Abca1*, *Apoa1* and *Pdk4* in the mucosa of the small intestine in wild-type mice but not in *PPAR*δ^*IEC-KO*^ mice (Fig. 4E,F). The expression of other genes involved in cholesterol transport such as *Abcg5* and *Npc1l1* and pro-inflammatory cytokine Tnf were not changed by GW501516 treatment in wild-type and *PPAR*δ^*IEC-KO*^ mice (Fig. 4E,F). Together these findings indicate that intestinal PPARδ is required for the increase in plasma levels of HDLc by PPARδ agonist treatment, whereas PPARδ elsewhere in the body does not significantly contribute to this effect.

## Discussion

In this study we investigated the role of intestinal PPARδ in energy metabolism and the development of metabolic syndrome using mice with an intestinal epithelial cell (IEC) specific deletion of PPARδ. Similar mice have previously been described and were shown to have a reduced incidence of azoxymethane-induced colon tumors^25^. Here we show that *PPAR*δ^*IEC-KO*^ mice display increased sensitivity to diet induced obesity and are unable to increase plasma HDLc levels after stimulation with the PPARδ specific agonist GW501516, indicating that intestinal PPARδ has an important role in the regulation of energy metabolism that cannot be compensated by PPARδ activation in other tissues.

The role of PPARδ in the intestine has mostly been studied for its anti-inflammatory effects and in the development of colorectal cancer (CRC)^26, 27^. PPARδ was originally implicated in CRC by its identification as a target of the adenomatous polyposis coli (APC) tumor suppressor, a key mediator in the development of CRC^28^. PPARδ expression is elevated in CRCs with a loss of function in the APC pathway and is repressed by expression of APC in CRC cells^28, 29^. Several studies using *Apc* ^*min*^ mice or chemically induced CRC have produced conflicting findings and currently there is no consensus on the role of PPARδ in in the development of CRC^29^.

Two independent whole body PPARδ knockout mouse models have been described^30, 31^. Both PPARδ knockout mouse models displayed an increased embryonic lethality due to a placental defect whereas surviving knockout animals were smaller and had reduced adiposity, especially at young age. Older, weight normalized PPARδ knockout mice, were found to display decreased metabolic activity and glucose intolerance when fed with a standard chow diet^32^. Contradicting results have been published on PPARδ whole body knockout mice challenged with a HFD, showing either increased obesity^33^ or a similar body weight gain but exaggerated glucose intolerance^32^. In the current study we found that *PPAR*δ^*IEC-KO*^ mice display increased sensitivity to diet-induced obesity and metabolic dysfunction characterized by insulin resistance and increased LDLc plasma levels.

The underlying mechanism by which intestinal PPARδ mediates its metabolic effects remains unclear since we did not find changes in food intake, activity or energy expenditure between wild-type and *PPAR*δ^*IEC-KO*^ mice. Previously, it has been shown that intestinal PPARδ plays a role in the stimulation of GLP-1 production in enteroendocrine L-cells, important for the preservation of β-cell morphology and function and, thereby, increased systemic insulin sensitivity^19^. In line with those observations, we found that the increase of proglucagon mRNA by a HFD was dependent on intestinal PPARδ. It remains unclear, however, whether a deficiency in the induction of GLP-1 in *PPAR*δ^*IEC-KO*^ mice contributes to the observed metabolic phenotype since plasma levels of GLP-1 after a glucose bolus were not affected by intestinal PPARδ. In addition to GLP-1, proglucagon mRNA processing in intestinal L-cells produces several other glucagon-related peptides including glucagon-like peptide-2 (GLP-2), oxyntomodulin (OXM) and glicentin^34^. The role of PPARδ in the regulation of these hormones and their contribution to the phenotypes of the *PPAR*δ^*IEC-KO*^ mice observed in this study, however, needs to be further investigated.

A role for PPARδ in Paneth cell differentiation has been described previously in PPARδ knockout mice and this was suggested to be associated with changes in the composition of intestinal microbiota^20^. In line with those findings we also found a reduction in the number of Paneth cells in chow fed mice but this was no longer observed after a HFD challenge. We also did not observe any changes in intestinal microbiota composition in PPAR δ^*IEC-KO*^ mice, suggesting that intestinal PPARδ alone is not a critical determinant in this regulation.

In addition to an increased sensitivity to diet-induced obesity, we show that *PPAR*δ^*IEC-KO*^ mice are unable to increase plasma HDLc levels after stimulation with the PPARδ specific agonist GW501516. There is a major interest in therapeutic strategies that raise the levels of serum HDLc as an approach to attenuate atherosclerosis by promoting reverse cholesterol transport (RCT) from peripheral tissues towards the liver^35^. In addition to improving RCT, PPARδ activation has also been shown to reduce intestinal cholesterol absorption via downregulation of Niemann-Pick C1-like 1 (NPC1L1) in the intestine, which may also contribute to its potential anti-atherogenic effects^21, 23^. Agonists for all three PPARs are known to enhance HDL biogenesis and this is mediated through transcriptional regulation of genes involved in HDL assembly including the ATP-binding cassette transporter A1 (ABCA1) which is rate limiting in this process^36^. ABCA1 mediates efflux of cholesterol and phospholipid from cells to lipid-free apoA-I, ultimately leading to the formation of nascent HDL particles. Mice lacking ABCA1 are unable to increase plasma HDLc in response to PPARδ activation, indicating that ABCA1 is essential in this process^21^. Approximately 70–80% of HDLc originates from the liver whereas 20–30% is produced by the intestine^37–39^. Although the ability of PPARs to increase HDL cholesterol levels has been typically attributed to their activation in the liver, it has recently been shown that PPARα-activation can also stimulate intestinal HDL-secretion *ex vivo* in human biopsies and Caco-2/TC7 cells^40^. Whether this is also the case for PPARδ, and to what extent the intestine contributes to the HDL-raising effects of PPAR ligands, remained unclear. Here we show that intestinal PPARδ is required for the stimulation of plasma HDLc levels by GW501516 and suggest that the role of hepatic PPARδ in HDL biogenesis is limited, at least at this dose of GW501516.

Taken together, our findings support intestinal-specific activation of PPARδ as a therapeutic approach for the treatment of dyslipidemia and other aspects of metabolic syndrome, while avoiding systemic toxicity.

## Materials and Methods

### Animals

Animals used in this study were male mice with an intestinal epithelial specific deletion of the *PPAR*δ gene (*PPAR* *δ* ^*IEC-KO*^) of a 99% C57BL/6 J genetic background between 6–16 wks of age. Mice harboring loxP sites on either side of exon 4 of the *PPAR*δ gene (B6.129S4-Ppard^tm1Rev^/J) have been described previously^31^. To generate *PPAR*δ^*IEC-KO*^ mice, *PPARδ* ^*lox/lox*^ mice were crossed with transgenic mice expressing Cre recombinase under the control of the villin promoter which is expressed in intestinal epithelial cells (IEC). Animals were housed in a light- and temperature-controlled facility (lights on from 7 a.m. to 7 p.m., 21 °C) with free access to water and standard chow (Arie Blok, The Netherlands, No. 4063 02), semi-synthetic low fat diet (LFD, 10% kcal from fat) (Open Source, The Netherlands, No. D12450J) or high-fat diet (HFD, 60% kcal from fat) (Open Source Diets, The Netherlands, No. D12492). Animal experiments were performed with the approval of the local Ethics Committee for Animal Experiments of the University of Groningen. All experiments were performed in accordance with relevant guidelines and regulations (including laboratory and biosafety regulations).

### Animal experiments

Mice were treated with 3 mg/kg GW501516 (Alexis/Enzo Life Sciences) or vehicle (0.5% methylcellulose) by daily oral gavage for 14 days. Mice were anesthetized with isoflurane and euthanized by cardiac puncture. Terminal blood samples were collected in EDTA-coated tubes. Tissues were collected and frozen in liquid nitrogen or processed for histology.

### Indirect calorimetry

Real-time metabolic analyses were performed using a Comprehensive Laboratory Animal Monitoring System (TSE systems GmbH, Bad Homburg, Germany). After a period of 24 h of acclimatization, CO_2_ production, O_2_ consumption, respiratory exchange ratio (RER), food intake and activity were determined for 48 h in individual mice.

### Glucose and insulin tolerance

Oral glucose tolerance tests (OGTT) were performed following oral administration of D-glucose at 2 g/kg body weight after a 6 h fast. Insulin tolerance tests (ITT) were performed following intraperitoneal (i.p.) administration of insulin (Novorapid, Novo Nordisk, Denmark) at 1 U/kg body weight after a 6 h fast. Blood glucose was monitored at 0, 15, 30, 60, 90 and 120 min after glucose or insulin administration using a OneTouch Ultra glucometer (Lifescan Inc, USA). Plasma insulin concentrations were determined using the ultra-sensitive mouse insulin ELISA kit from Crystal Chem (Cat. 90080, USA). Plasma GLP1 levels were determined following oral administration of D-glucose at 2 g/kg body weight after a 6 h fast. The samples were immediately treated with a DPP4 inhibitor (Merck Millipore Cat. DPP4, USA) and measured using the Active GLP1 Kit from MSD (ver. 2, Cat. K150JWC-1).

### Fat balance

For determination of the fat balance, food intake was recorded and feces were collected over a period of 72 h. Fecal pellets were freeze-dried and mechanically homogenized. Lipids were extracted from the samples, hydrolyzed, and methylated as described previously^41^. The resulting fatty acid methyl esters of LCFA were analyzed and quantified by gas chromatography, using heptadecanoic acid as an internal standard. The fat absorption coefficient (%) was calculated by subtracting the fecal fat output (g/day) from the fat intake (g/day), divided by the fat intake (g/day) multiplied by 100%.

### BA and NS analysis

Total BA and NS concentrations were determined in feces as previously described^42, 43^. Briefly, BA profiles were determined after deconjugation and extraction with commercially available Sep-Pak-C18 (Mallinckrodt Baker, The Netherlands) cartridges and conversion to their methylester/trimethylsilyl derivatives. NS in feces were saponified and extracted with hexane. BA and NS were analyzed using capillary gas chromatography.

### Lipid and lipoprotein analysis

Pooled plasma samples were subjected to fast protein liquid chromatography (FPLC) gel filtration using a Superose 6HR10/300GL column (GE Healthcare, Uppsala, Sweden) as described^44^. Individual fractions of 0,5 ml plasma diluted in PBS were analyzed for cholesterol and triglyceride concentrations by spectrophotometry using commercially available kits (Roche Diagnostics, Mannheim, Germany). HDLc and LDLc levels in plasma were determined using a commercially available kit (Abcam, Cambrige, UK). Hepatic lipids were extracted according to Bligh & Dyer^45^. TGs were determined using the Trig/GB (Triglycerides glycerol blanked) kit (Roche #11877771).

### Gene expression analysis

Total RNA was isolated from intestinal mucosa or liver using Tri reagent (Life Technologies, USA) and reverse transcribed into cDNA using M-MLV, random primers and dNTPs according to standard procedures. For quantitative PCR (qPCR), cDNA was amplified using Hi-ROX SensiMix™ SYBR green (Bioline, London, UK) and StepOnePlus™ Real-Time PCR System (Applied Biosystems, CA, USA). Primers used for qPCR are listed in Supplementary Table 3. 36b4 was used as the house-keeping gene in all PCR analyses and the ∆∆Ct method was used for quantification.

### Histological analysis and immunohistochemistry

For microscopic examination, tissues were fixed in 4% phosphate-buffered formalin, embedded in paraffin, sectioned at 4 μm, and stained with haematoxylin and eosin (H&E). Histological scoring was performed in an unbiased manner by two board certified veterinary pathologists (L.H and A.d.B.). Hepatic steatosis and inflammation were graded in H&E stained liver sections by using an adapted version of the NAS scoring system for NAFLD developed by Kleiner *et al*.^46^.

### Analysis of microbiota

For microbiota analysis, samples were collected on chow and after treatment with GW501516 or HFD. The middle and distal third of the small intestine, cecum and colon including content was removed and homogenized in lysis buffer. Bacterial DNA from the homogenate was isolated using the QIAamp DNA Stool Mini Kit (Qiagen, Valencia, CA), quantified by spectrophotometry and 50 ng (15 ng respectively for universal bacterial primer) of DNA was amplified by RT-PCR using the SensiMix™ SYBR® Hi-ROX Kit (Bioline, Taunton, MA) and bacterial group-specific primers for 16S as previously described^47^.

### Statistical analysis

Statistics were performed using the GraphPad Prism 5.00 software package (GraphPad Software, San Diego, CA, USA). Significance was determined using the nonparametric Mann Whitney U-test when comparing two groups or the Kruskal-Wallis H-test when comparing more groups. In case of significant Kruskal-Wallis test, Dunns posthoc test was performed. All values are given as means ± SEM unless stated otherwise. Significance was indicated as *P < 0.05, **P < 0.01, ***P < 0.001.

## Electronic supplementary material

Supplementary information

## References

[1] Evans RM, Barish GD, Wang Y-X (2004). PPARs and the complex journey to obesity. Nat. Med..

[2] Glass CK, Saijo K (2010). Nuclear receptor transrepression pathways that regulate inflammation in macrophages and T cells. Nat. Rev. Immunol..

[3] Hashimoto T (2000). Defect in Peroxisome Proliferator-activated Receptor alpha -inducible Fatty Acid Oxidation Determines the Severity of Hepatic Steatosis in Response to Fasting. J. Biol. Chem..

[4] Vu-Dac N (1995). Fibrates increase human apolipoprotein A-II expression through activation of the peroxisome proliferator-activated receptor. J. Clin. Invest..

[5] Pawlak M, Lefebvre P, Staels B (2015). Molecular mechanism of PPARα action and its impact on lipid metabolism, inflammation and fibrosis in non-alcoholic fatty liver disease. J. Hepatol..

[6] Tontonoz P, Spiegelman BM (2008). Fat and Beyond: The Diverse Biology of PPARγ. Annu. Rev. Biochem.

[7] Vazquez-Carrera M (2016). Unraveling the Effects of PPARbeta/delta on Insulin Resistance and Cardiovascular Disease. Trends Endocrinol. Metab..

[8] Wang YX (2003). Peroxisome Proliferator Activated Receptor [delta] Activates Fat Metabolism to Prevent Obesity. Cell.

[9] Wang YX (2004). Regulation of muscle fiber type and running endurance by PPARdelta. PLoS Biol..

[10] Leibowitz MD (2000). Activation of PPARdelta alters lipid metabolism in db/db mice. FEBS Lett.

[11] Lee C-H (2003). Transcriptional repression of atherogenic inflammation: modulation by PPARdelta. Science.

[12] Oliver WR (2001). A selective peroxisome proliferator-activated receptor delta agonist promotes reverse cholesterol transport. Proc. Natl. Acad. Sci. USA.

[13] Sprecher DL (2007). Triglyceride:high-density lipoprotein cholesterol effects in healthy subjects administered a peroxisome proliferator activated receptor delta agonist. Arterioscler. Thromb. Vasc. Biol..

[14] Narkar Va (2009). AMPK and PPARdelta agonists are exercise mimetics. Cell.

[15] Bays HE (2011). MBX-8025, a novel peroxisome proliferator receptor-delta agonist: lipid and other metabolic effects in dyslipidemic overweight patients treated with and without atorvastatin. J. Clin. Endocrinol. Metab..

[16] Iwaisako K (2012). Protection from liver fibrosis by a peroxisome proliferator-activated receptor δ agonist. Proc. Natl. Acad. Sci. USA.

[17] Mackenzie LS, Lione L (2013). Harnessing the benefits of PPARbeta/delta agonists. Life Sci..

[18] Bookout AL (2006). Anatomical profiling of nuclear receptor expression reveals a hierarchical transcriptional network. Cell.

[19] Daoudi M (2011). PPARβ/δ activation induces enteroendocrine L cell GLP-1 production. Gastroenterology.

[20] Varnat F (2006). PPARbeta/delta regulates paneth cell differentiation via controlling the hedgehog signaling pathway. Gastroenterology.

[21] van der Veen JN (2005). Reduced cholesterol absorption upon PPARdelta activation coincides with decreased intestinal expression of NPC1L1. J. Lipid Res..

[22] Vrins CLJ (2009). Peroxisome proliferator-activated receptor delta activation leads to increased transintestinal cholesterol efflux. J. Lipid Res..

[23] Briand F (2009). Both the peroxisome proliferator-activated receptor δ agonist, GW0742, and ezetimibe promote reverse cholesterol transport in mice by reducing intestinal reabsorption of HDL-Derived cholesterol. Clin. Transl. Sci.

[24] Olson EJ, Pearce GL, Jones NP, Sprecher DL (2012). Lipid effects of peroxisome proliferator-activated receptor-delta agonist GW501516 in subjects with low high-density lipoprotein cholesterol: Characteristics of metabolic syndrome. Arterioscler. Thromb. Vasc. Biol..

[25] Zuo X (2009). Targeted genetic disruption of peroxisome proliferator-activated receptor-delta and colonic tumorigenesis. J. Natl. Cancer Inst..

[26] Peters JM, Hollingshead HE, Gonzalez FJ (2008). Role of peroxisome-proliferator-activated receptor beta/delta (PPARbeta/delta) in gastrointestinal tract function and disease. Clin. Sci. (Lond)..

[27] Beyaz S (2016). High-fat diet enhances stemness and tumorigenicity of intestinal progenitors. Nature.

[28] He TC, Chan TA, Vogelstein B, Kinzler KW (1999). PPARdelta is an APC-regulated target of nonsteroidal anti-inflammatory drugs. Cell.

[29] Park BH, Vogelstein B, Kinzler KW (2001). Genetic disruption of PPARdelta decreases the tumorigenicity of human colon cancer cells. Proc. Natl. Acad. Sci. USA.

[30] Peters JM (2000). Growth, adipose, brain, and skin alterations resulting from targeted disruption of the mouse peroxisome proliferator-activated receptor beta(delta). Mol. Cell. Biol..

[31] Barak Y (2002). Effects of peroxisome proliferator-activated receptor delta on placentation, adiposity, and colorectal cancer. Proc. Natl. Acad. Sci. USA.

[32] Lee C-H (2006). PPARdelta regulates glucose metabolism and insulin sensitivity. Proc. Natl. Acad. Sci. USA.

[33] Luquet S (2003). Peroxisome proliferator-activated receptor delta controls muscle development and oxidative capability. FASEB J..

[34] Bataille D, Dalle S (2014). The forgotten members of the glucagon family. Diabetes Res. Clin. Pract..

[35] Kingwell BA, Chapman MJ, Kontush A, Miller NE (2014). HDL-targeted therapies: progress, failures and future. Nat. Rev. Drug Discov..

[36] Chinetti G (2001). PPAR-alpha and PPAR-gamma activators induce cholesterol removal from human macrophage foam cells through stimulation of the ABCA1 pathway. Nat. Med.

[37] Francis GA, Knopp RH, Oram JF (1995). Defective removal of cellular cholesterol and phospholipids by apolipoprotein A-I in Tangier disease. J. Clin. Invest..

[38] Timmins JM (2005). Targeted inactivation of hepatic Abca1 causes profound hypoalphalipoproteinemia and kidney hypercatabolism of apoA-1. J. Clin. Invest..

[39] Brunham, L. R. *et al*. Intestinal ABCA1 directly contributes to HDL biogenesis *in vivo*. **116**, 1052–1062 (2006). 10.1172/JCI27352 PMC1401485 16543947

[40] Colin S (2013). Activation of intestinal peroxisome proliferator-activated receptor-α increases high-density lipoprotein production. Eur. Heart J..

[41] Muskiet FA, van Doormaal JJ, Martini IA, Wolthers BG, van der SW (1983). Capillary gas chromatographic profiling of total long-chain fatty acids and cholesterol in biological materials. J.Chromatogr..

[42] Setchell KDR, Worthington J (1982). A rapid method for the quantitative extraction of bile acids and their conjugates from serum using commercially available reverse-phase octadecylsilane bonded silica cartridges. Clin. Chim. Acta.

[43] Gerhardt KO, Gehrke CW (1977). Gas-Liquid Chromatography of Fecal Neutral Steroids. J. Chromatogr..

[44] Nijstad N (2009). Scavenger receptor BI-mediated selective uptake is required for the remodeling of high density lipoprotein by endothelial lipase. J. Biol. Chem..

[45] Bligh E, Dyer WJ (1959). A rapid method of total lipid extraction and purification. Can. J. Biochem. Physiol..

[46] Kleiner DE (2005). Design and validation of a histological scoring system for nonalcoholic fatty liver disease. Hepatology.

[47] Wouthuyzen-Bakker M (2012). Effect of antibiotic treatment on fat absorption in mice with cystic fibrosis. Pediatr Res..

