# Supplementary material for: Intestinal PPARδ protects against diet-induced obesity, insulin resistance and dyslipidemia
Source: Sci Rep. 2017 Apr 12;7:846. doi: 10.1038/s41598-017-00889-z (PMC5429805; doi:10.1038/s41598-017-00889-z)
Supplement: Supplementary file 1 — Supplementary information [file 41598_2017_889_MOESM1_ESM.pdf]

Intestinal PPARδ protects against diet-induced obesity, insulin resistance and dyslipidemia

Marcela Doktorova[1*], Irene Zwarts[1*], Tim van Zutphen[1], Theo H. van Dijk[2], Vincent W. Bloks[1], Liesbeth Harkema[3], Alain de Bruin[1,3], Michael Downes[4], Ronald M. Evans[4], Henkjan J. Verkade[1] and Johan W. Jonker[1#]

[1]Section of Molecular Metabolism and Nutrition, Department of Pediatrics and [2]Department of Laboratory Medicine, University of Groningen, University Medical Center Groningen, Hanzeplein 1, 9713 GZ Groningen, The Netherlands. [3]Dutch Molecular Pathology Center, Faculty of Veterinary Medicine, Utrecht University, Yalelaan 1, 3584 CL Utrecht, The Netherlands. [4]Howard Hughes Medical Institute and Gene Expression Laboratory, Salk Institute for Biological Studies, 10010 North Torrey Pines Road, La Jolla, California 92037, USA. [#]To whom correspondence should be addressed. E-mail: j.w.jonker@umcg.nl. [*]These authors contributed equally to this work.

Supplementary data

Supplementary Table 1

	Proximal		Middle		Distal	
**LFD**	wild-type	*PPARδ*$^{IEC\text{-}KO}$	wild-type	*PPARδ*$^{IEC\text{-}KO}$	wild-type	*PPARδ*$^{IEC\text{-}KO}$
**Villus length (µm)**	398 ± 14	412 ± 73	202 ± 26	224 ± 27	197 ± 13	190 ± 22
**Crypt depth (µm)**	58 ± 5	64 ± 5	69 ± 8	62 ± 3	71 ± 11	67 ± 10
**Inflammation scoring**	0	0	0	0	0	0
**HFD**	wild-type	*PPARδ*$^{IEC\text{-}KO}$	wild-type	*PPARδ*$^{IEC\text{-}KO}$	wild-type	*PPARδ*$^{IEC\text{-}KO}$
**Villus length (µm)**	410 ± 35	383 ± 66	266 ± 70	329 ± 79	212 ± 66	242 ± 40
**Crypt depth (µm)**	71 ± 9	64 ± 8	69 ± 13	69 ± 4	67 ± 12	73 ± 14
**Inflammation scoring**	0	0	0	0	0	0

Supplementary Table 1. Histopathologic analysis of HE stained intestinal sections of wild-type and *PPARδ*$^{IEC\text{-}KO}$ mice after a LFD (n = 3) or a HFD (n = 8). Values are presented as mean ± SD. Inflammation scoring: 0 = no inflammation, 1 = mild inflammation, 2 = moderate inflammation, 3 = severe inflammation.

Supplementary Table 2

HFD	wild-type	$PPAR\delta^{IEC\text{-}KO}$
**Sum of steatosis**	0.5 [0-2]	2 [0-3] *
**Lobular inflammation**	0 [0-1]	0.75 [0-2]
**Ballooning**	0 [0-1]	0 [0-1]
**NAFLD activity score**	0.5 [0-3.5]	3 [0-5] *

Supplementary Table 2. Liver composition of wild-type and $PPAR\delta^{IEC\text{-}KO}$ mice fed a HFD. Values are presented as median; [range] (n = 8). Steatosis grade 0= <5%; 1 = 5-33%; 2 = 33-66%; 3 = >66%. Lobular inflammation 0= none; 1 = <2 foci per 200x; 2 = 2-4 foci. Ballooning 0 = none; 1 = few; 2 = prominent ballooning. NAS = NAFLD activity score (sum of steatosis + lobular inflammation + ballooning). *Significant difference between wild-type and $PPAR\delta^{IEC\text{-}KO}$ mice ($p<0.05$).

Supplementary Table 3

Gene	Forward primer 5’ --- 3’	Reverse primer 5’ --- 3’
**36b4**	CTG TTG GCC AAT AAG GTG CC	GGA GGT CTT CTC GGG TCC TA
**Abca1**	CCC AGA GCA AAA AGC GAC TC	GGT CAT CAT CAC TTT GGT CCT TG
**Abcg5**	CTC CTC GCC TAC GTG CTA CA	GAT ACA AGC CCA GAG TCC AAT AAC A
**Apoa1**	CCC AGT CCC AAT GGG ACA	CAG GAG ATT CAG GTT CAG CTG TT
**Cre**	GCA TTA CCG GTC GAT GCA ACG AGT G	GAA CGC TAG AGC CTG TTT TGC ACG TTC
**Gcg**	CAA GAG GAA CCG GAA CAA CAT T	CCT GGC CCT CCA AGT AAG AA
**Npc1l1**	GAG AGC CAA AGA TGC TAC TAT CTT CA	CCC GGG AAG TTG GTC ATG
**Pdk4**	GCA TTT CTA CTC GGA TGC TCA TG	CCA ATG TGG CTT GGG TTT CC
**Ppard**	CTC AAT GGG GGA CCA GAA CA	AAG GGG AGG AAT TCT GGG AGA
**Tnf**	GTA GCC CAC GTC GTA GCA AAC	AGT TGG TTG TCT TTG AGA TCC ATG

Supplementary Table 3: qPCR primer sequences.

Supplementary Figure 1

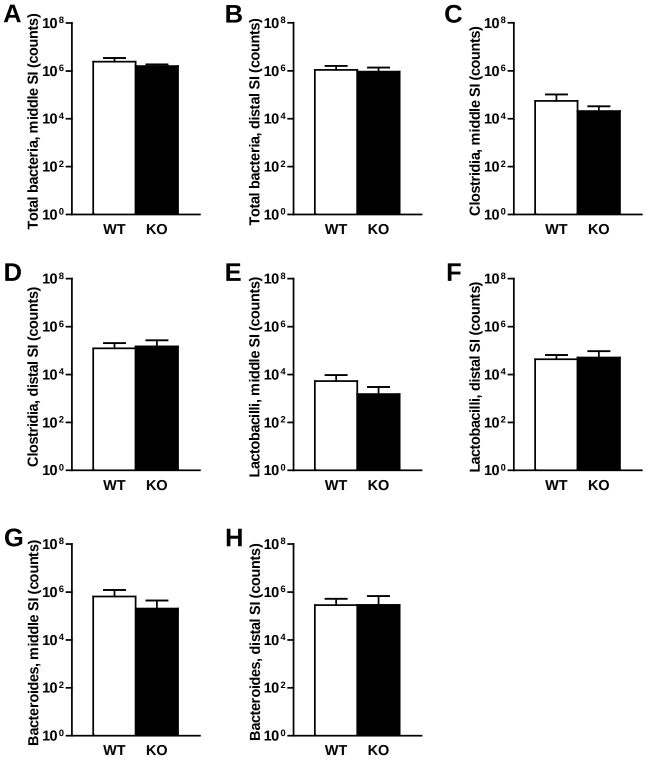

Supplementary Figure 1. Intestinal PPARδ does not regulate bacterial colonization of small intestine. Bacterial counts of **(A,B)** total bacteria and **(C,D)** bacterial groups of Clostridium; **(E,F)** Lactobacilli and **(G,H)** Bacteroides in middle and distal part of the intestine of wild-type and *PPARδ*$^{IEC\text{-}KO}$ mice. (n = 4-5)

Supplementary Figure 2

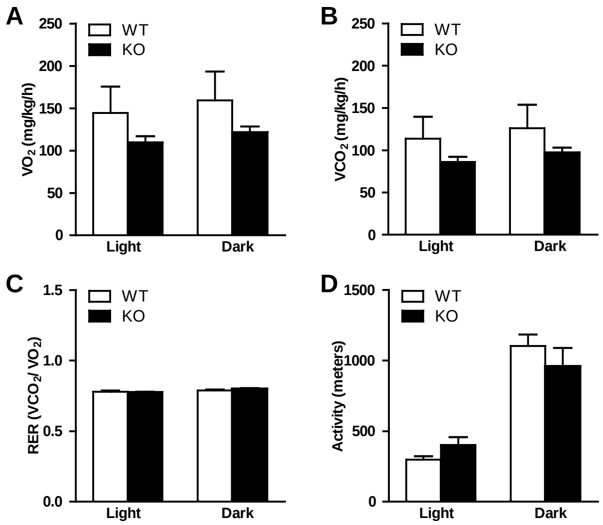

Supplementary Figure 2. Effect of a HFD on metabolic parameters in *PPARδ*$^{IEC\text{-}KO}$ and wild-type mice. (A) Oxygen consumption (VO_2); **(B)** Carbon dioxide production (VCO_2); **(C)** Respiratory exchange ratio (RER) and **(D)** Activity in *PPARδ*$^{IEC\text{-}KO}$ mice and wild-type littermates (n = 4-6) after a HFD challenge.
